# Low antibody prevalence against *Bacillus cereus* biovar *anthracis* in Taï National Park, Côte d'Ivoire, indicates high rate of lethal infections in wildlife

**DOI:** 10.1371/journal.pntd.0005960

**Published:** 2017-09-21

**Authors:** Fee Zimmermann, Susanne M. Köhler, Kathrin Nowak, Susann Dupke, Anne Barduhn, Ariane Düx, Alexander Lang, Hélène M. De Nys, Jan F. Gogarten, Roland Grunow, Emmanuel Couacy-Hymann, Roman M. Wittig, Silke R. Klee, Fabian H. Leendertz

**Affiliations:** 1 Robert Koch Institute, P3: “Epidemiology of Highly Pathogenic Microorganisms", Seestraße 10, Berlin, Germany; 2 Robert Koch Institute, ZBS 2: Centre for Biological Threats and Special Pathogens, Highly Pathogenic Microorganisms, Seestraße 10, Berlin, Germany; 3 Department of Biology, McGill University, Montreal, QC, Canada; 4 Primatology Department, Max Planck Institute for Evolutionary Anthropology, Deutscher Platz 6, Leipzig, Germany; 5 Department of Virology, LANADA/LCVB, Bingerville, Côte d'Ivoire; 6 Taï Chimpanzee Project, Centre Suisse de Recherches Scientifiques, Abidjan 01, Côte d’Ivoire; University of California Davis, UNITED STATES

## Abstract

*Bacillus cereus* biovar *anthracis (Bcbva)* is a member of the *B*. *cereus* group which carries both *B*. *anthracis* virulence plasmids, causes anthrax-like disease in various wildlife species and was described in several sub-Saharan African rainforests. Long-term monitoring of carcasses in Taï National Park, Côte d’Ivoire, revealed continuous wildlife mortality due to *Bcbva* in a broad range of mammalian species. While non-lethal anthrax infections in wildlife have been described for *B*. *anthracis*, nothing is known about the odds of survival following an anthrax infection caused by *Bcbva*. To address this gap, we present the results of a serological study of anthrax in five wildlife species known to succumb to *Bcbva* in this ecosystem. Specific antibodies were only detected in two out of 15 wild red colobus monkeys (*Procolobus badius*) and one out of 10 black-and-white colobus monkeys (*Colobus polykomos*), but in none of 16 sooty mangabeys (*Cercocebus atys*), 9 chimpanzees (*Pan troglodytes verus*) and 9 Maxwell’s duikers (*Cephalophus maxwellii*). The combination of high mortality and low antibody detection rates indicates high virulence of this disease across these different mammalian species.

## Author summary

Anthrax is a deadly zoonosis, predominantly known to affect wild and domestic herbivores. It has long been assumed that the disease is exclusively caused by *B*. *anthracis*, but recently another member of the *B*. *cereus* group, *Bacillus cereus* biovar *anthracis (Bcbva)*, was found to carry both *B*. *anthracis* virulence plasmids. *Bcbva* causes anthrax-like disease in wildlife throughout sub-Saharan Africa and was shown to be an important cause of wildlife mortality in Taï National Park, Cote d’Ivoire, affecting a broad range of mammalian species. While mortality data has routinely been collected in the area for decades, it remains unknown whether non-lethal *Bcbva* exposure occurs. We therefore conducted a serological study in four primate and one duiker species in which *Bcbva*-related fatalities were previously documented. Frequent non-lethal exposure should result in a high antibody seroprevalence within wildlife populations, while high lethality would result in low antibody seroprevalence. We found that antibody detection rates were low, suggesting that *Bcbva* infections in these species are likely often lethal.

## Introduction

Anthrax is a zoonosis occurring worldwide, characterized by septicemia and sudden death, mainly in herbivores. The disease is regularly observed in arid and savanna ecosystems, where animals ingest bacterial spores from soil while grazing [1–3]. In the past anthrax was thought to be exclusively caused by bacteria of the clonal *Bacillus anthracis* clade within the *Bacillus cereus* group. Anthrax-like disease, caused by *Bacillus cereus* biovar *anthracis* (*Bcbva*), was first reported in 2001 from Taï National Park (TNP), Côte d’Ivoire, where it caused sudden death in chimpanzees [4]. While *Bcbva* carries the two *B*. *anthracis* virulence plasmids, pXO1 and pXO2, it is more closely related to other members of the *B*.*cereus* group at the chromosomal level [5,6]. The fatalities in TNP represented the first observation of anthrax-like disease in wild non-human primates and in a rainforest ecosystem [4]. Subsequently, *Bcbva* was found to be widespread throughout tropical forests of sub-Saharan Africa, including Cameroon, the Central African Republic, the Democratic Republic of the Congo and Liberia [6–8]. In TNP, continuous carcass monitoring from 2001 to 2015 showed *Bcbva* to be a major driver of wildlife mortality; *Bcbva* was the cause of death for over 40% of carcasses found by researchers in this tropical ecosystem [8]. The high prevalence of anthrax-like disease observed at TNP is exceptional, even when compared to other African national parks where anthrax caused by *B*. *anthracis* is endemic and considered common [9,10].

**Fig 1:**
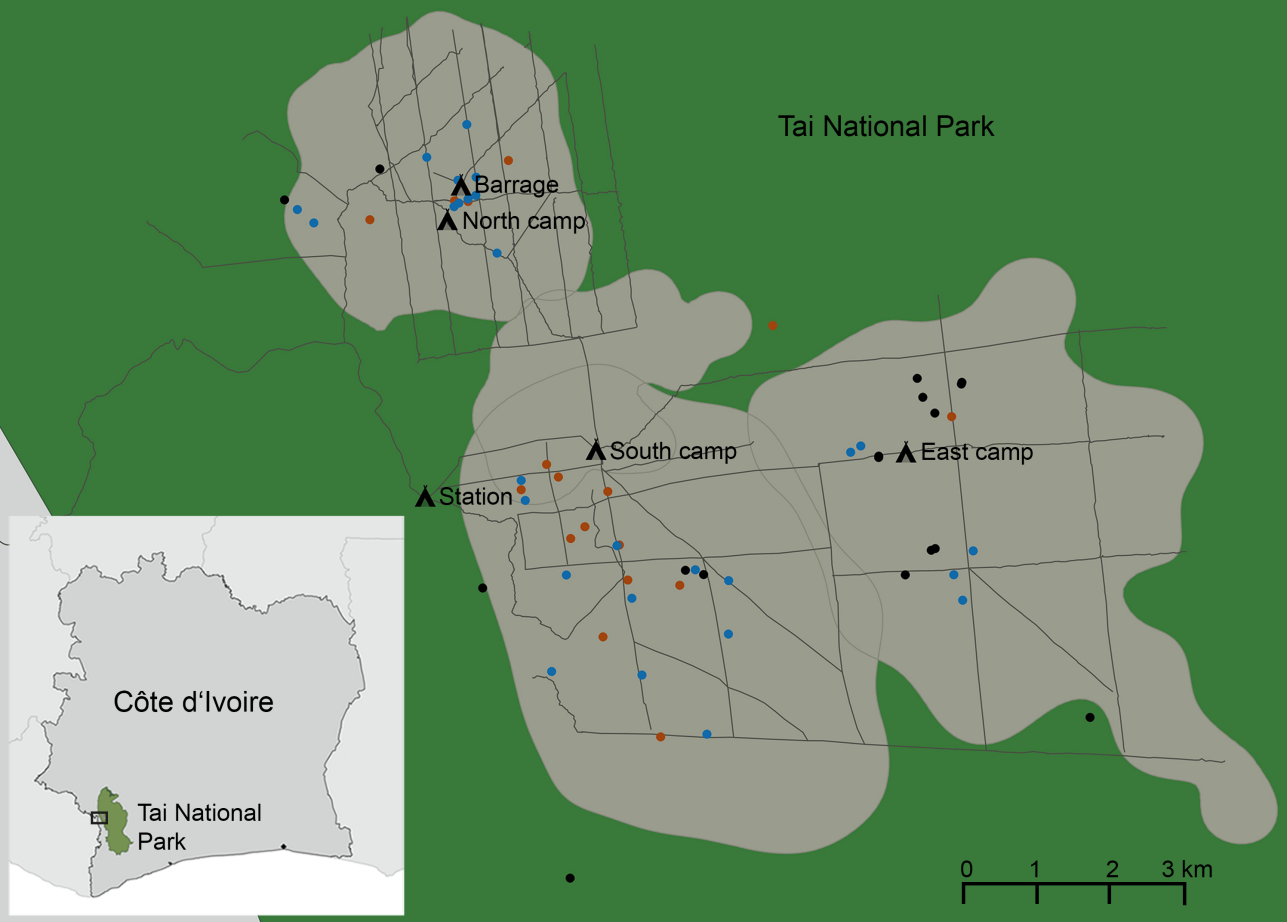
*Bcbva* positive necropsies in Taï National Park from 2006 to 2015. Taï National Park is located in the south-west of Côte d’Ivoire near the Liberian border (0°15’– 6°07’N, 7°25’– 7°54’W). The box in the overview map indicates the area enlarged in the big map. Carcass monitoring has revealed continuous occurrence of *Bcbva* in the research area (marked in gray in the big map) of the Taï Chimpanzee Project. All tested serological samples were collected in this area between 2006 and 2015. The 62 out of 139 (45%) carcasses that tested positive for *Bcbva* in this period are indicated in the map. Blue dots show duiker carcasses, red dots monkey carcasses and black dots chimpanzee carcasses. The figure has been created by the authors of the manuscript with the freely available software QGIS. Shape files for Africa were freely available at http://maplibrary.org/library/index.htm.

Anthrax outbreaks in African savanna national parks, caused by *B*. *anthracis*, are usually wavelike and primarily affect a few (ungulate) species at a time [1,3,11,12]. This contrasts with the situation at TNP where a broad range of mammalian hosts succumb to the disease simultaneously [8]. To date, lethal *Bcbva* infections have been documented in chimpanzees (*Pan troglodytes verus*), six species of monkeys (*Cercocebus atys*, *Cercopithecus campbelli*, *Cercopithecus diana*, *Cercopithecus petaurista*, *Procolobus badius* and *Colobus polykomos*), duikers (*Cephalophus* spp.), mongooses (fam. Herpestidae) and porcupines (fam. Hystricidae). Fatalities were observed year-round and were distributed evenly across the area of research (Fig 1). Culturable *Bcbva* was detected in 5% of randomly caught carrion flies, which highlights the persistent nature of *Bcbva* in TNP and its broad distribution throughout the sampled region of the park [8].

Gross and histopathology of *Bcbva* cases in wildlife are comparable to that of anthrax cases caused by *B*. *anthracis*. In small animal models *Bcbva* exhibits a similar virulence to what has been observed for *B*. *anthracis*, previously [4,13]. Unfortunately nothing is known about the likelihood of survival following infection with *Bcbva* for rainforest fauna living in the wild. Monkeys and chimpanzees were observed to die within hours of the onset of disease symptoms by the primatologists of the Taï Chimpanzee Project. The rapid mortality following the onset of symptoms could potentially be a product of the generally suppressed expression of signs of weakness in wild animals [14]; we do not know the incubation time for *Bcbva*, though available evidence suggests *Bcbva* is highly virulent [13]. To date, anthrax surveillance in TNP was largely carried out by carcass monitoring [8]. However, carcass detection is heavily biased in its detection probability for larger–bodied species, especially in areas with dense vegetation, and it only provides information about infections with a lethal outcome [8,15,16].

Serological approaches offer a complementary means of understanding disease ecology; in combination with carcass monitoring data, insights can be made about the susceptibility of different species to a disease. Carcass monitoring and serological studies from savanna ecosystems suggest, that herbivorous ungulates are generally highly susceptible to anthrax, while carnivores appear to be more resistant [1]. This is supported by high levels of seropositivity observed in most carnivore and omnivore savanna species, combined with low observed fatality rates, which suggests regular non-fatal exposure [10,16,17]. On the other hand, the relatively low seroprevalence in combination with high mortality rates, which are observed in savanna herbivores, suggests these species are more susceptible to the disease [10,16]. Bagamian et al. [18] used such logic to call for the broad combination of postmortem data with serological surveillance, specifically in non-savanna ecosystems, to further assess the dynamics of anthrax disease [16].

Here we present a serological investigation in TNP in the context of existing mortality records. We focused on five species for which we previously detected *Bcbva* associated fatalities [8]: herbivorous red colobus monkeys (3/30 carcasses *Bcbva* positive) and black-and-white colobus monkeys (1/5 carcasses *Bcbva* positive), omnivorous sooty mangabeys (11/23 carcasses *Bcbva* positive) and chimpanzees (31/55 carcasses *Bcbva* positive) and opportunistically scavenging omnivorous Maxwell’s duikers (26/40 carcasses *Bcbva* positive). We examined serum, plasma or whole blood samples and tested for antibodies against the anthrax protective antigen (PA) and lethal factor (LF) to characterize the *Bcbva* antibody detection rate.

## Materials and methods

### Study location (TNP)

TNP is an evergreen rainforest located in the south-west of Côte d’Ivoire (0°15’– 6°07’N, 7°25’– 7°54’W). The climate of TNP is sub-equatorial with two rainy seasons (major: August-October, minor: March-June) and a total average annual rainfall of 1800 mm. While the Upper Guinea Forest belt once stretched from Ghana to Sierra Leone, TNP is the largest remaining section today, covering an area of 3300 km^2^, and is surrounded by a 200 km^2^ buffer zone. Almost 1000 species of vertebrates have been described in the TNP ecosystem, and the park was awarded UNESCO Natural World Heritage status in 1982 [19,20]. This work has been performed in the research area of the Taï Chimpanzee Project that has studied the local habituated chimpanzee groups since 1979 [19].

### Serum collection

#### Monkeys

Samples of red colobus monkeys, black-and-white-colobus monkeys and sooty mangabeys were collected between 2006 and 2015, resulting in a total of 41 monkey samples (S1 Table). Monkeys were anesthetized using a combination of ketamine (5mg/kg) and medetomidine (0.05mg/kg), administered intramuscularly via blowpipe (Telinject GmbH, Dudenhofen, Germany) or dart gun (Dan- Inject, Borkop, Denmark). Induction took 5–10 min and anesthesia lasted for 30–40 min. Blood samples were collected in EDTA coated tubes. After antagonization with an intramuscular injection of 0.25mg/kg atipamezole, recovery took 60–120 min. Animals were not left before they could climb without observable difficulties and returned to their social group. Blood samples were centrifuged at 3000 rpm for 10 min upon return to our forest laboratory, separated into plasma, buffy coat and erythrocytes and subsequently stored in liquid nitrogen. Samples were transported on dry ice and conserved at -80°C for long-term storage.

#### Duikers

Sampling of Maxwell’s duikers was performed in 2013 and 2016 (S1 Table). A total of nine duikers were trapped using the night-time net capture technique described by Newing [21], which is based on the animals freezing when stunned with a strong flashlight. Following capture with the net capture technique, animals were initially anesthetized using medetomidine (0.1 mg/kg), ketamine (4.0 mg/kg) and midazolam (0.1 mg/kg). Because the level of anesthesia reached with this combination of anesthetics was rather deep for our needs, dosages were adapted to 0.07 mg/kg medetomidine and 2.5 mg/kg ketamine with slightly increased midazolam (0.17 mg/kg). This combination yielded appropriate anesthetic depth for our sampling needs. Induction took 5–10 min and animals were anesthetized for 30–50 min. The same sampling protocol described above for monkeys was used for duikers. After antagonization with atipamezole, animals fully recovered within 20–90 minutes. A large well-trained team is needed for this method for capturing duikers and further optimization might be possible (e.g., combining dazzling with a flashlight with GPS marked darts for anesthesia).

#### Chimpanzees

One chimpanzee serum sample taken under anesthesia during an emergency surgery in a chimpanzee with air-sacculitis was available from 2009 [22]. A further eight whole blood samples were obtained during necropsies of freshly deceased animals that died in outbreaks of respiratory disease in 2004, 2006 and 2009 that tested negative for *Bcbva* in qPCR (S1 Table) [23,24].

### Ethics statement

All wildlife samples were collected with permission of the research ministries of Côte d’Ivoire and ethical approval of the Ivorian Office of National Parks, which reviewed the study design (permits Nr. 048/MESRS/DGRSIT/KCS/TM and 90/MESRS/DGRSIT/mo). Samples have been exported with the required CITES (Convention on International Trade in Endangered Species of Wild Fauna and Flora) permits. The study was approved by the Centre Suisse de Recherche Scientifique en Côte d’Ivoire and the Laboratoire National de la Pathologie Animale, Bingerville, Côte d’Ivoire.

All chimpanzee samples originated from free-ranging chimpanzees. Samples were collected from chimpanzees that had died of natural reason in outbreaks of respiratory disease by our team of veterinarians routinely investigating wildlife mortality in TNP [23,24]. In one case samples were obtained from an individual on whom surgery had to be performed due to a life threatening infection [22]. No chimpanzee was anesthetized or touched for the sole purpose of sample collection.

Human sera used as controls in this study were donated by the authors of the study themselves and were anonymized immediately after sample donation. Humans were not vaccinated against anthrax to serve as controls in this study, but had received anthrax vaccinations in the past due to their work in anthrax endemic areas. All human sera were donated by adults after giving written informed consent, and the use of human sera in this study was approved by the ethics committee of Charité-Universitätsmedizin Berlin.

### Serological approach

No standardized approaches are available to investigate anthrax seroprevalence in wildlife. Thus, all samples were tested for antibodies against the anthrax protective antigen (PA, Quadratech Diagnostics, Surrey, UK) using an in-house ELISA and an in-house Western Blot and for antibodies against anthrax lethal factor (LF, Quadratech Diagnostics) using an in-house Western Blot. Assays are described in detail below.

Testing for PA and LF does not allow for a discrimination between classical *B*. *anthracis* and *Bcbva*, as both pathogens produce the typical anthrax toxins [25]. However, during our extensive carcass monitoring over the last 15 years, each of the 81 anthrax cases that were detected in TNP was caused by *Bcbva*. This was shown by qPCR screening for the *Bcbva* specific genomic island IV, isolation and subsequent whole-genome sequencing [8]. Therefore, in the TNP ecosystem antibodies generated against PA and LF likely originate exclusively from exposure to *Bcbva*.

Human positive and negative controls were used for all monkey and chimpanzee assays as no species-specific controls were available. Negative controls were selected from a set of available human sera of unvaccinated donors, which were unreactive in Western Blot against PA and LF. Two of these sera that were representative of the range for PA-negative human sera in PA ELISA were chosen as negative controls and included on each ELISA test-plate under the same conditions as the samples for inter-plate comparison. A positive control serum from a hyper-immunized human donor was included as an 8-step log_2_ serial dilution curve (starting concentration: 1 in 4000) with repetitious reactivity and accurate results on every test-plate.

For duiker assays, a negative control was available from a red forest duiker (*Cephalophus natalensis*, courtesy of Berlin zoo), which was unreactive in PA and LF Western Blot. A pool of goats vaccinated with the *B*. *anthracis* Sterne spore live vaccine [26] (courtesy of Dr. W. Beyer) was used as a positive control in the same fashion as stated for the positive human control (starting concentration: 1 in 1000). No specific conjugated antibodies were available for any of the species tested. For primate samples, polyvalent goat anti-human horseradish peroxidase (HRP) labeled conjugate (Dianova, Hamburg, Germany) was used, as described previously [27,28]. For duikers, we tested the reactivity of duiker serum with different commercially available conjugates from the Bovidae family (sheep, cow, goat) in a comparative dot blot approach with logarithmic duiker serum dilutions starting at 1:10. We found that polyvalent rabbit anti-goat HRP labeled conjugate (Dianova, Hamburg, Germany) was the most suitable commercially available conjugate for duiker samples.

### ELISA

PA ELISA was performed as described by Hahn et al., with slight modifications [29,30]. Briefly, each well of high-binding microtiter plates (Maxisorp Nunc, Sigma Aldrich, Munich, Germany) was coated with 0.1 μg of recombinant PA in PBS at 4°C overnight. Wells were washed with phosphate-buffered saline containing 0.02% (v/v) Tween 20 (PBS-Tween) and blocked with 5% skimmed milk powder in PBS-Tween. Samples and negative controls were diluted 1:500 in blocking solution and incubated in duplicate together with the positive and negative controls for 2 hours at room temperature. Secondary antibodies were used in a concentration of 1:10000 and 1:4000 (previously evaluated and adjusted) for humanoid and duiker assays, respectively. Plates were developed in the dark with 100 μl of TMB SeramunBlau fast (Seramun Diagnostics GmbH, Heidesee, Germany) substrate per well for 10 min and stopped with 100 μl H_2_SO_4_ (2M). Absorbance was measured at 450nm (reference wavelength 620nm) using a Tecan Sunrise 96-well-reader (Tecan Group Ltd., Männedorf, Switzerland). The mean of the negative controls plus two times their standard deviation (SD) was set as the cut-off value for each plate (S1 Table).

### Western blot

For the PA and LF Western Blot assay, 380 ng of purified recombinant PA or LF diluted in 125 μl of phosphate-buffered saline (PBS) were blotted onto an Immobilion-P PVDF-Membrane (Merck, Darmstadt, Germany) after running in a 12% agarose gel. Then 3 mm stripes (approx. 25 per gel) were cut from the membrane and samples and controls were added in a dilution of 1:1000 in the dilution buffer containing tris-buffered saline with 0.05% Tween (TBS-Tween) and 3% powdered milk. Samples were incubated at room temperature for one hour. Goat anti-human HRP conjugate was added to primate samples and human controls in a 1:10000 dilution in the dilution buffer (1:8000 for LF Western Blot). For duiker samples and goat/duiker controls, rabbit anti-goat HRP conjugate was diluted 1:4000 in the dilution buffer (1:8000 for LF Western Blot). The conjugate was left to incubate for one hour. Reactions were detected with precipitating peroxidase substrate TMB SeramunBlau prec (Seramun Diagnostics GmbH, Heidesee, Germany) after 10 min of incubation.

## Results

A total of 59 serum, plasma or whole blood samples from five different TNP wildlife species were tested, mainly representing primates. All samples originated in an area where *Bcbva* is known to be endemic and where 40% of carcasses detected in the past were tested positive for *Bcbva* by qPCR, mostly confirmed by bacterial isolation, histology and whole genome sequencing [8] (Fig 1). Despite the continuous occurrence of the disease in the research area, we found that antibody detection rates in wildlife were low.

For red colobus monkeys (n = 15) and black-and-white colobus monkeys (n = 10), one sample for each species was clearly positive in PA ELISA and could be confirmed in PA and LF Western Blot. One more red colobus sample reacted in PA Western Blot, but in none of the other assays. For duikers (n = 9) four samples were borderline positive in PA ELISA, but none of those were confirmed in PA or LF Western Blot. None of the sooty mangabey (n = 16) or chimpanzee (n = 9) samples reacted in PA ELISA, PA or LF Western Blot. Results are presented in detail in Table 1 and the supplementary S1 Table.

**Table 1:** Results overview.

species	n	PA ELISA positive	PA WB positive	LF WB positive	observed seroprevalence (95% CI)	positive control	negative control	conjugate
Western red colobus monkey (*Procolobus badius*)	15	1	2	1	13% (2–42%)	AVA vaccinated human	human	goat α human
Western black-and-white colobus monkey (*Colobus polykomos*)	10	1	1	1	10% (0–46%)	AVA vaccinated human	human	goat α human
Sooty mangabey (*Cercocebus atys*)	16	0	0	0	0% (0–24%)	AVA vaccinated human	human	goat α human
Chimpanzee (P*an troglodytes verus*)	9	0	0	0	0% (0–37%)	AVA vaccinated human	human	goat α human
Maxwell's duiker (*Cephalophus maxwelli*)	9	4	0	0	0% (0–37%)	Sterne vaccinated goat	Red forest duiker (*Cephalophus natalensis*) from zoo	rabbit α goat

Results for PA ELISA, PA and LF Western Blot in each tested species. Observed seroprevalence includes all PA Western Blot positive animals. The 95% confidence intervals for seroprevalence were approximated with the *prop*.*test* function in R [31].

## Discussion

We found low seroprevalence for antibodies against anthrax, despite *Bcbva* being responsible for a substantial proportion of disease-induced mortality in each of the tested species. Specific antibodies against PA (in Western Blot) were only detected in three samples with two individuals (a red colobus monkey and a black-and-white colobus monkey) also showing OD values in ELISA in the range of the positive control and a positive result in LF Western Blot. While the sample set presented here may not appear extensive in comparison to datasets collected in savanna ecosystems, it represents the largest available collection of serum from TNP wildlife. The small sample sizes cause considerable uncertainty, when calculating species-specific seroprevalence (Table 1), but the data imply low overall seroprevalence in TNP wildlife.

In many anthrax serological studies, samples are broadly screened with an ELISA alone and positive samples are then confirmed by Western Blot. However, serological anthrax surveillance of wildlife populations is often complicated by a lack of species-specific controls, conjugates, and validated assays. This makes definition of fixed ELISA cut-off values difficult, which complicates the interpretation of results. In our study, human conjugates and controls were used to investigate primate samples and goat conjugates and vaccinated goats as positive controls were used for duiker assays. For duikers, a negative control from a related species, a red forest duiker, was available from a zoo. However, using zoo animals as controls for wildlife can be potentially misleading, because these animals are often unexposed to many other pathogens and symbionts circulating in an ecosystem that may increase immunological background. Determining an ELISA cut-off with the same approach as used for primates (mean of negative controls + 2*SD) classified four of the duiker samples as positive, as the zoo negative control had an extremely low OD value. However, none of these samples that were reactive in ELISA were subsequently confirmed in Western Blot (for neither PA nor LF). While a species-specific negative control was available in this case, the ELISA cut-off was likely artificially low due to the different immunological background in animals of diverging origin. In contrast, for red colobus monkeys a sample classified as negative in ELISA was found to be positive in the more sensitive PA Western Blot. Otherwise, results from ELISA and Western Blot approaches were largely congruent. The OD amplitudes of animals that tested positive in Western Blot showed higher ODs than the rest of their sampling group (if not higher than the cut-off). ELISA testing of negative control animals from zoos or related species can provide a guideline for the interpretation of wildlife ELISA results, but our results highlight the problems associated with using strict ELISA cut-offs as a stand-alone criterion for non-validated wildlife assays and the importance of using both ELISA and Western Blot screening approaches.

While the finding of only three seropositive samples could be interpreted as a consequence of low exposure to *Bcbva*, our previous studies have shown that *Bcbva* cases occur in TNP throughout the year [8]. The finding of culturable *Bcbva* in carrion flies randomly caught in the study area further indicated the occurrence of *Bcbva* cases even when no carcasses were detected. In fact, comparison of whole genome sequences of *Bcbva* isolated from carrion flies and simultaneously found carcasses revealed carcass monitoring data only reflects a fraction of the actual *Bcbva* mortality in this dense rainforest environment [8].

The low seroprevalence observed from live animals together with the previously described high mortality rates in these species (5) support the hypothesis that sublethal infections exist, but at very low rates. It has been shown previously that sublethal (*B*. *anthracis*) anthrax infections do not only occur in carnivores. Different degrees of anthrax seroprevalence have been reported for herbivores in African savannas [10,17,32]. The finding of anthrax antibodies in wildebeest (*Conochaetes taurinus mearnsi*) (19%) and buffalo (*Syncerus caffer*) (46%) in the Serengeti showed that not all herbivores succumb to infection and that susceptibility can vary widely between herbivore species [10]. Significant seropositivity was also observed in zebra (*Equus quagga*) in Etosha National Park [32]. Our study in TNP included arboreal species (red colobus monkeys and black-and-white colobus monkeys) and species that live and feed at least partly on the forest floor (chimpanzees, sooty mangabeys and duikers). Two individuals of the arboreal monkey species tested showed high OD values in PA ELISA and were confirmed in PA and LF Western Blot. These strong immune reactions might suggest that these animals recently survived an infection. One potential scenario for infection of arboreal monkeys could be cutaneous infection by a vector, e.g., biting flies, as observed previously in cattle and humans [33,34] or mechanical transmission onto canopy foods by carrion flies. Anthrax positive carrion flies have been found in the forest canopy at TNP lending some credence to the latter possibility [8], though experimental studies are needed to confirm the plausibility of such infection routes. Unfortunately, serological testing for antibodies against PA and LF cannot differentiate infection with subsequent recovery from low dose exposure with seroconversion. Seroconversion through intestinal absorption of PA and other toxin components was suggested for scavengers by Turnbull et al. [35] and must also be considered when investigating a newly detected pathogen in an ecosystem where transmission pathways are largely unknown. It is striking that for the three species suffering the highest observed *Bcbva* related mortality rates in the park and that are likely regularly exposed to soil containing anthrax spores (chimpanzees, mangabeys and duikers), not a single animal showed a measurable immune response against anthrax that would suggest exposure to *Bcbva*.

Despite the ubiquitous presence of *Bcbva* in TNP causing high amounts of *Bcbva* related mortality, the majority of animals tested here show no antibodies against this disease. These results suggest that systemic infections with *Bcbva* are generally fatal in the five tested species and that this disease could potentially pose a serious threat to conservation efforts in the region.

## Supporting information

S1 Table Individual serological data (ELISA, WB). Table containing information on the origin of individual samples and results for ELISA and Western Blot testing. Given are the mean measured ELISA OD_450nm_ values for all samples tested (sorted by species) and their respective values for the assay internal negative control. ELISA measurements were conducted in a 1:500 dilution and the mean of the blanks was subtracted. To assess the quality of the measurements, the coefficient of variation was calculated and readings were rerun if it was higher than 0.2. The human positive control for primate testing was titrated in an 8 step log_2_ serial dilution on the plate with a starting concentration of 1:4000 and yielded a titre of 512000. For duikers, each plate was validated through inclusion of a pooled positive control serum from goats vaccinated with the *B*. *anthracis* Sterne spore live vaccine. This was titrated in an 8 step log_2_ serial dilution on the plate with a starting concentration of 1:1000 and yielded a titre of 128000. LF and PA Western Blot were performed with 1:1000 dilutions for all samples and controls.
(XLSX)
